# Injection drug use among street-involved youth in a Canadian setting

**DOI:** 10.1186/1471-2458-9-171

**Published:** 2009-06-03

**Authors:** Thomas Kerr, Brandon DL Marshall, Cari Miller, Kate Shannon, Ruth Zhang, Julio SG Montaner, Evan Wood

**Affiliations:** 1 British Columbia Centre for Excellence in HIV/AIDS, St. Paul's Hospital, Vancouver, British Columbia, Canada; 2 Department of Medicine, University of British Columbia, Vancouver, British Columbia, Canada; 3 Faculty of Health Sciences, Simon Fraser University, Burnaby, Canada

## Abstract

**Background:**

Street-involved youth contend with an array of health and social challenges, including elevated rates of blood-borne infections and mortality. In addition, there has been growing concern regarding high-risk drug use among street-involved youth, in particular injection drug use. We undertook this study to examine the prevalence of injection drug use and associated risks among street-involved youth in Vancouver, Canada.

**Methods:**

From September 2005 to November 2007, baseline data were collected for the At-Risk Youth Study (ARYS), a prospective cohort of street-recruited youth aged 14 to 26 in Vancouver, Canada. Using multiple logistic regression, we compared youth with and without a history of injection.

**Results:**

The sample included 560 youth among whom the median age was 21.9 years, 179 (32%) were female, and 230 (41.1%) reported prior injection drug use. Factors associated with injection drug use in multivariate analyses included age ≥ 22 years (adjusted odds ratio [AOR] = 1.18, 95% CI: 1.10–1.28); sex work involvement (AOR = 2.17, 95% CI: 1.35–3.50); non-fatal overdose (AOR = 2.10, 95% CI: 1.38–3.20); and hepatitis C (HCV) infection (AOR = 22.61, 95% CI: 7.78–65.70).

**Conclusion:**

These findings highlight an alarmingly high prevalence of injection drug use among street-involved youth and demonstrate its association with an array of risks and harms, including sex work involvement, overdose, and HCV infection. These findings point to the need for a broad set of policies and interventions to prevent the initiation of injection drug use and address the risks faced by street-involved youth who are actively injecting.

## Background

Street-involved youth are increasingly common in North American cities and are a population at heightened risk for an array of health and social problems [1]. In Canada and the United States, it is estimated that between 4% and 7% of youth between the ages of 14 and 26 are absolutely, periodically, or temporarily without safe and stable shelter [2,3]. Since the definition of "street youth" is often extended to individuals who may be housed but who are heavily involved in the street economy [4], the proportion of youth who fall into this category may be significantly greater.

Compared to the general population, street-involved youth contend with a variety of health-related risks and challenges [1], including elevated rates of mental illness [5], blood-borne infections [5-7], sexually transmitted infections [8], and violence [9,10]. Recent evidence from Montreal, Canada suggests that street-involved youth, in comparison to the general population, face an 8 to 11 times higher risk of mortality [11].

There have also been persistent concerns regarding high-risk substance use among street-involved youth in settings throughout the world [12-14]. In particular, in light of the associated risks for human immunodeficiency virus (HIV) and hepatitis C infection (HCV), increasing attention has been paid to the initiation of injection drug use within this population. This has led to calls for an updating of the risk hierarchy so that the prevention of injection drug use is given greater attention [15]. However, the prevalence of injecting and associated harms among street-involved youth has not been studied in many urban areas in North America [13,16,17], and, outside of Montreal [18], little is known about the epidemiology of injection drug use among street-involved youth in Canada. Past estimates suggest there are at any time upwards of 150,000 young people in Canada who are absolutely, periodically or temporarily without shelter [2]. However, there remain many challenges associated with estimating the size of this population given the transitory nature of street youth and lack of reliable counts of street youth (e.g., Census data, etc).

Vancouver, Canada has been the site of co-occurring epidemics of injection drug use, HIV, overdose, and HCV. However, while much study has been devoted to adult injection drug users (IDU) in this setting, little is known about the prevalence and associated risks of injection drug use among the city's growing street youth population [19]. Although reliable estimates are lacking, it has been suggested that there are between 500 to 1000 street youth living on Vancouver streets each day [20]. While an increasing number of studies have described the predictors of the initiation and cessation of injection drug use [18,21-23], there have been few studies that have compared the characteristics of drug-using street youth who have and have not injected. Therefore, we undertook this study to assess the prevalence and related risks and harms of injection drug use among a cohort of drug-using street-involved youth in Vancouver.

## Methods

The At-Risk Youth Study (ARYS) is a prospective cohort study of Vancouver street-involved youth that has been described in detail previously [19]. Briefly, participants were recruited through snowball sampling and extensive street-based outreach. Persons were eligible for the study if they were between 14 and 26 years of age, had used illicit drugs other than or in addition to marijuana in the past 30 days, and provided informed consent. At baseline and at semi-annual follow-up visits, participants complete an interviewer-administered questionnaire and provide blood samples for HIV and hepatitis C (HCV) serology. The questionnaire elicits demographic data and information regarding injection and non-injection illicit drug use, HIV risk behaviors, income generation, encounters with police, health service utilization, and sexual activity. The questionnaire is based on a previous instrument developed for adult drug users, and has been found to reliably identify factors associated with HIV infection, mortality, and other harms [24-26]. All participants received a monetary stipend of $20 after each visit. The study has been approved by the University of British Columbia/Providence Health Care Research Ethics Board.

The present study was conducted to evaluate the prevalence of injection drug use and related risks and harms among street-involved youth who completed baseline study visits between September 2005 and November 2007. Although ARYS is a prospective cohort study, given the low incidence of initiation into injection drug use, a longitudinal study of injection drug use initiation among ARYS participants was not possible at this time. The primary outcome of interest was reporting any history of injection drug use (i.e., lifetime prevalence of injection drug use). Other self-reported variables of interest were selected based on a review of the documented harms associated with injection drug use, and included: age; gender; Aboriginal ethnicity; history of incarceration; history of sex work involvement (defined as exchanging sex for money, drugs, shelter, or gifts); history of drug dealing; currently having a warrant for one's arrest; and history of non-fatal overdose. Variable definitions were consistent with prior studies involving the ARYS cohort [27,28]. Serologic outcomes considered included HIV- and HCV-positive status.

As a first step, we conducted a univariate analyses in which those who did and did not report a history of injection drug use were compared using Pearson's Chi-square test and the Wilcoxon rank test. Fisher's exact test was used when one or more of the cells contained values less than or equal to five. As a second step, a multivariate model was then prepared using an *a priori *defined statistical approach whereby variables that were *p *< 0.05 in univariate analyses were included in a fixed logistic regression model. Variables that remained significant at *p *< 0.05 were regarded as factors significantly associated with the outcome of interest. All statistical analyses were performed using SAS software version 9.1 (SAS, Cary, NC), and all *p*-values are two sided.

## Results

In total, 560 participants were recruited and included in the present analyses. Of these participants, 179 (32%) were female and 131 (23.4%) were of Aboriginal ancestry. The median age was 21.9 years (interquartile range = 19.8–23.9). In total, 230 (41.1%) participants reported injection drug use. Factors associated with injection drug use in univariate analyses are presented in Table 1 and include: age ≥ 22 year (odds ratio [OR] = 2.67, 95% confidence interval [CI]: 1.89–3.79, *p *< 0.001); history of incarceration (OR = 2.04, 95% CI: 1.31–3.18, *p *< 0.001); sex work involvement (OR = 2.86, 95% CI: 1.89–4.32, *p *< 0.001); drug dealing (OR = 1.61, 95% CI: 1.10–2.42, *p *= 0.023); having a warrant for one's arrest (OR = 1.56, 95% CI: 1.03–2.33, *p *= 0.037); non-fatal overdose (OR = 2.76, 95% CI: 1.92–3.97, *p *< 0.001); and HCV infection (OR = 29.51, 95% CI: 10.55–82.53, *p *< 0.001).

**Table 1:** Factors associated with injection drug use among street-involved youth (n = 560).

**Characteristic**	**Yes n = 230 (41.1%)**	**No n = 330 (58.9%)**	**Odds Ratio (95% CI)**	***p *- value**
**Median age**				

≥ 22 years	82 (35.6)	197 (59.7)	2.67 (1.89–3.79)	<0.001

< 22 years	148 (64.4)	133 (40.3)		

**Gender**				

Female	67 (29.1)	112 (33.9)	0.80 (0.56–1.15)	0.233

Male	163 (70.9)	218 (66.1)		

**Aboriginal ethnicity**				

Yes	51 (22.2)	80 (24.2)	0.89 (0.60–1.33)	0.570

No	179 (77.8)	250 (75.8)		

**Incarceration**				

Yes	197 (85.7)	246 (74.5)	2.04 (1.31–3.18)	<0.001

No	33 (14.3)	84 (25.5)		

**Sex work**				

Yes	74 (32.2)	47 (14.2)	2.86 (1.89–4.32)	<0.001

No	156 (67.8)	283 (85.8)		

**Drug dealing**				

Yes	187 (81.3)	241 (73.0)	1.61 (1.10–2.42)	0.023

No	43 (18.7)	89 (27.0)		

Warrants*				

Yes	60 (26.0)	61 (18.5)	1.56 (1.03–2.33)	0.037

No	170 (74.0)	269 (81.5)		

**Overdose**				

Yes	107 (46.5)	79 (23.9)	2.76 (1.92–3.97)	<0.001

No	123 (53.5)	251 (76.1)		

**HCV-positive†**				

Yes	62 (26.9)	4 (1.2)	29.51(10.55–82.53)	<0.001

No	167 (72.6)	318 (96.4)		

**HIV-positive‡**				

Yes	7 (3.0)	8 (2.4)	1.64 (0.58–4.61)	0.427

No	221 (96.1)	319 (96.7)		

* Warrants refers to having a warrant for arrest

† HCV refer to hepatitis C virus

‡ HIV refers to human immunodeficiency virus

Values do not add up to 100% due to missing tests results

As shown in Table 2, factors found to be independently associated with injection drug use in multivariate logistic regression analyses included: age = 22 years (adjusted odds ratio [AOR] = 1.18, 95% CI: 1.10–1.28, *p *< 0.001); sex work involvement (AOR = 2.17, 95% CI: 1.35–3.50, *p *= 0.001); non-fatal overdose (AOR = 2.10, 95% CI: 1.38–3.20, *p *< 0.001); and HCV infection (AOR = 22.61, 95% CI: 7.78–65.70, *p *< 0.001).

**Table 2:** Logistic regression analysis of factors associated with injection drug use among street-involved youth (n = 560).

**Variable**	**Adjusted Odds Ratio (AOR)**	**95% Confidence Interval (95% CI)**	***p *- value**
**Age**			

(≥ 22 vs. < 22 years)	1.18	(1.10 – 1.28)	<0.001

**Incarceration**			

(yes vs. no)	1.54	(0.88 – 2.70)	0.124

**Sex work**			

(yes vs. no)	2.17	(1.35 – 3.50)	0.001

**Overdose**			

(yes vs. no)	2.10	(1.38 – 3.20)	<0.001

**Drug dealing**			

(yes vs. no)	1.20	(0.73 – 1.97)	0.472

Warrants*			

(yes vs. no)	1.18	(0.73 – 1.93)	0.498

**HCV-positive**			

(yes vs. no)	22.61	(7.78 – 65.70)	<0.001

* Warrants refers to having a warrant for arrest

## Discussion

In the present analysis, we found that injection drug use was common among street-involved youth in Vancouver, with approximately 40% of the cohort reporting a history of injection drug use. In a multivariate analysis, reporting a history of injection drug use was associated with older age, sex work involvement, non-fatal overdose, and hepatitis C infection.

The high prevalence of injection drug use among street-involved youth in Vancouver is slightly lower than observed in Montreal, Canada (41% versus 60%) [18], although somewhat higher than observed in some US cities [17]. Injection drug use among street-involved youth in this study was associated with several markers of risk, including sex work involvement. This is consistent with previous studies from various settings demonstrating a relationship between injection drug use and sex work among street-involved youth [29-32], studies which have also suggested that the co-occurrence of injection drug use and sex work places street-involved youth at elevated risk for HIV infection and other sexually transmitted infections. The association between sex work and injection drug use observed in the present study is also consistent with previous research demonstrating that injection drug users (IDU) frequently resort to high-risk income generating activities, such as sex work [33], to support their drug use and basic survival needs [34], and that sex work is often linked to drug scene exposure [35]. However, previous studies from our setting have also suggested a strong relationship between the initiation of sex work among street-involved youth and childhood emotional and sexual abuse [36]. While the forces that prompt the co-occurrence of sex-work involvement and injection drug use will require further elucidation, the finding of an association between sex work and injection drug use among street-involved youth in this settings is of immediate concern, given evidence suggesting that sex work involvement is a strong predictor of premature mortality among young IDU in Vancouver [25].

Injection drug use was also associated with non-fatal overdose in this study. This is consistent with other studies indicating a high rate of prior non-fatal overdose among IDU [37,38] and a relationship between elevated risk for overdose and injecting as a route of drug administration [39]. Although non-fatal overdose among IDU is typically associated with heroin use [39], it is notable that crystal methamphetamine use is highly prevalent among street-involved youth in Vancouver and has been associated with non-fatal overdose among this population and adult IDU [27,40].

The finding that injection drug use was strongly associated with an elevated risk for HCV infection among youth in this study is consistent with a previous study of street-involved youth in Montreal and is not surprising, given the high prevalence of HCV infection among adult IDU in Vancouver [41] and the fact that injection drug use is an efficient mode of HCV transmission. Indeed, previous studies undertaken within North America suggest that many young IDU acquire HCV soon after initiating injecting, with the estimated time to HCV infection among this population being 1 to 2 years [42,43].

These findings reinforce previous calls for an updating of the risk prevention hierarchy so that the transition to injection drug use receives greater attention [15]. Indeed, studies have shown that the risk for acquisition of blood-borne infections is high after the onset of injecting [44], and therefore interventions that prevent the initiation of injecting among street-involved youth are needed, including those that target individuals (e.g., addiction treatment), as well as those that address the broader social, structural, and environmental determinants of injection drug use [45]. Unfortunately, few evidence-based approaches of this kind have been described. Given that older age (> 22 years) was associated with injection drug use in this study, future efforts should include delivering prevention interventions to high-risk street youth not yet exposed to injecting. Further, because these findings indicate that street-involved youth are at heightened risk for an array of harms through both drug use (e.g., overdose and HCV infection) and related activities (e.g., sex work involvement), interventions that reduce harm among street-involved youth who have taken up injecting are also needed. These include conventional harm reduction interventions [46], such as youth-friendly needle exchange programs, but also social and structural interventions that aim to mediate the risk environment of youth who inject drugs and exchange sex, including mobile service delivery and socio-legal reforms that facilitate safe sex work settings [47,48].

Our study has several limitations. First, like most other cohort studies involving high-risk youth, ARYS is not a random sample, and therefore our study findings may not be generalize well to the larger population of street youth in Vancouver. Further, given the differences across settings, including differences in the types of drugs used in different urban environments, our findings may not generalize well to street youth in other settings. Second, we relied on several measures of self-report, and therefore some response biases may have affected our results. In particular, we may have underestimated some sensitive behaviors and experiences, including injection drug use and sex work involvement. Lastly, this study design is cross-sectional in nature, and therefore causal relationships cannot be inferred.

## Conclusion

In summary, we found a high prevalence of injection drug use among street-involved youth in Vancouver. Importantly, injection drug use was associated with several markers of elevated risk and harm, including sex work involvement and overdose, as well as hepatitis C infection. These findings point to the need for continued efforts to reduce the initiation into injection use, as well as efforts to minimize the risks and harms experienced by street-involved youth who already inject.

## Competing interests

The authors declare that they have no competing interests.

## Authors' contributions

TK and EW designed the study. RZ and TK conducted the statistical analyses. TK and EW drafted the manuscript and incorporated all suggestions. All authors made significant contributions to the conception and design of the analyses, interpretation of the data, and drafting of the manuscript, and all authors approved the final manuscript.

## Pre-publication history

The pre-publication history for this paper can be accessed here:



## References

[B1] Boivin JF, Roy E, Haley N, Galbaud du Fort G (2005). The health of street youth: a Canadian perspective. Can J Public Health.

[B2] Public Health Agency of Canada (2006). Street youth in Canada: findings from enhanced surveillance of Canadian street youth, 1999–2003.

[B3] Ringwalt CL, Greene JM, Robertson M, McPheeters M (1998). The prevalence of homelessness among adolescents in the United States. American Journal of Public Health.

[B4] DeMatteo D, Major C, Block B, Coates R, Fearon M, Goldberg E, King SM, Millson M, O'Shaughnessy M, Read SE (1999). Toronto street youth and HIV/AIDS: prevalence, demographics, and risks. J Adolesc Health.

[B5] Nyamathi AM, Christiani A, Windokun F, Jones T, Strehlow A, Shoptaw S (2005). Hepatitis C virus infection, substance use and mental illness among homeless youth: a review. AIDS.

[B6] Roy E, Haley N, Leclerc P, Cedras L, Weber AE, Claessens C, Boivin JF (2003). HIV incidence among street youth in Montreal, Canada. AIDS.

[B7] Roy E, Haley N, Leclerc P, Boivin JF, Cedras L, Vincelette J (2001). Risk factors for hepatitis C virus infection among street youths. CMAJ.

[B8] Tyler KA, Whitbeck LB, Chen X, Johnson K (2007). Sexual health of homeless youth: prevalence and correlates of sexually transmissible infections. Sexual Health.

[B9] Martin I, Palepu A, Wood E, Li K, Montaner J, Kerr T (2009). Violence among street-involved youth: the role of methamphetamine. European Addiction Research.

[B10] Stewart AJ, Steiman M, Cauce AM, Cochran BN, Whitbeck LB, Hoyt DR (2004). Victimization and posttraumatic stress disorder among homeless adolescents. Journal of the American Academy of Child and Adolescent Psychiatry.

[B11] Roy E, Haley N, Leclerc P, Sochanski B, Boudreau JF, Boivin JF (2004). Mortality in a cohort of street youth in Montreal. JAMA.

[B12] Howard J (1993). Taking a chance on love: risk behaviour of Sydney street youth. Journal of Paediatrics and Child Health.

[B13] Gleghorn AA, Marx R, Vittinghoff E, Katz MH (1998). Association between drug use patterns and HIV risks among homeless, runaway, and street youth in northern California. Drug and Alcohol Dependence.

[B14] Raffaelli M (1999). Homelessness and working street youth in Latin America: a developmental review. Interamerican Journal of Psychology.

[B15] Vlahov D, Fuller CM, Ompad DC, Galea S, Des Jarlais DC (2004). Updating the Infection Risk Reduction Hierarchy: Preventing Transition into Injection. J Urban Health.

[B16] Molnar BE, Shade SB, Kral AH, Booth RE, Watters JK (1998). Suicidal behavior and sexual/physical abuse among street youth. Child Abuse & Neglect.

[B17] Lifson AR, Halcon LL (2001). Substance abuse and high-risk needle-related behaviors among homeless youth in Minneapolis: implications for prevention. J Urban Health.

[B18] Roy E, Boudreau JF, Leclerc P, Boivin JF, Godin G (2007). Trends in injection drug use behaviors over 10 years among street youth. Drug and Alcohol Dependence.

[B19] Wood E, Stoltz JA, Montaner JS, Kerr T (2006). Evaluating methamphetamine use and risks of injection initiation among street youth: the ARYS study. Harm Reduction Journal.

[B20] McCarthy B (1995). On the Streets – Youth in Vancouver.

[B21] Roy É, Haley N, Leclerc P, Cédras L, Blais L, Boivin J-F (2003). Drug injection among street youths in montreal: Predictors of initiation. Journal of Urban Health.

[B22] Ompad DC, Ikeda RM, Shah N, Fuller CM, Bailey S, Morse E, Kerndt P, Maslow C, Wu Y, Vlahov D (2005). Childhood sexual abuse and age at initiation of injection drug use. American Journal of Public Health.

[B23] Steensma C, Boivin JF, Blais L, Roy E (2005). Cessation of injecting drug use among street-based youth. J Urban Health.

[B24] Wood E, Spittal P, Li K, Kerr T, Miller CL, Hogg RS, Montaner JS, Schechter MT (2004). Inability to Access Addiction Treatment and Risk of HIV Infection Among Injection Drug Users. Journal of Acquired Immune Deficiency Syndromes (1999).

[B25] Miller CL, Kerr T, Strathdee SA, Li K, Wood E (2007). Factors associated with premature mortality among young injection drug users in Vancouver. Harm Reduction Journal.

[B26] Tyndall MW, Currie S, Spittal P, Li K, Wood E, O'Shaughnessy MV, Schechter MT (2003). Intensive injection cocaine use as the primary risk factor in the Vancouver HIV-1 epidemic. Aids.

[B27] Werb D, Kerr T, Lai C, Montaner J, Wood E (2008). Nonfatal overdose among a cohort of street-involved youth. J Adolesc Health.

[B28] Wood E, Stoltz JA, Zhang R, Strathdee SA, Montaner JS, Kerr T (2008). Circumstances of first crystal methamphetamine use and initiation of injection drug use among high-risk youth. Drug and Alcohol Review.

[B29] Weber AE, Boivin JF, Blais L, Haley N, Roy E (2002). HIV risk profile and prostitution among female street youths. J Urban Health.

[B30] Haley N, Roy E, Leclerc P, Boudreau JF, Boivin JF (2004). HIV risk profile of male street youth involved in survival sex. Sexually Transmitted Infections.

[B31] Swart-Kruger J, Richter LM (1997). AIDS-related knowledge, attitudes and behaviour among South African street youth: reflections on power, sexuality and the autonomous self. Soc Sci Med.

[B32] Shakarishvili A, Dubovskaya LK, Zohrabyan LS, St Lawrence JS, Aral SO, Dugasheva LG, Okan SA, Lewis JS, Parker KA, Ryan CA (2005). Sex work, drug use, HIV infection, and spread of sexually transmitted infections in Moscow, Russian Federation. Lancet.

[B33] Cusick L (2006). Widening the harm reduction agenda: From drug use to sex work. International Journal of Drug Policy.

[B34] DeBeck K, Shannon K, Wood E, Li K, Montaner J, Kerr T (2007). Income generating activities of people who inject drugs. Drug and Alcohol Dependence.

[B35] Strathdee SA, Philbin MM, Semple SJ, Pu M, Orozovich P, Martinez G, Lozada R, Fraga M, de la Torre A, Staines H (2008). Correlates of injection drug use among female sex workers in two Mexico-U.S. border cities. Drug and Alcohol Dependence.

[B36] Stoltz JA, Shannon K, Kerr T, Zhang R, Montaner JS, Wood E (2007). Associations between childhood maltreatment and sex work in a cohort of drug-using youth. Soc Sci Med.

[B37] Kerr T, Fairbairn N, Tyndall M, Marsh D, Li K, Montaner J, Wood E (2007). Predictors of non-fatal overdose among a cohort of polysubstance-using injection drug users. Drug and Alcohol Dependence.

[B38] Darke S, Ross J, Hall W (1996). Overdose among heroin users in Sydney, Australia: I. Prevalence and correlates of non-fatal overdose. Addiction (Abingdon, England).

[B39] Darke S, Hall W (2003). Heroin overdose: research and evidence-based intervention. J Urban Health.

[B40] Fairbairn N, Wood E, Stoltz JA, Li K, Montaner J, Kerr T (2008). Crystal methamphetamine use associated with non-fatal overdose among a cohort of injection drug users in Vancouver. Public health.

[B41] Wood E, Kerr T, Stoltz J, Qui Z, Zhang R, Montaner JS, Tyndall MW (2005). Prevalence and correlates of hepatitis C infection among users of North America's first medically supervised safer injection facility. Public Health.

[B42] Hagan H, Des Jarlais DC, Stern R, Lelutiu-Weinberger C, Scheinmann R, Strauss S, Flom PL (2007). HCV synthesis project: preliminary analyses of HCV prevalence in relation to age and duration of injection. Int J Drug Policy.

[B43] Miller CL, Johnston C, Spittal PM, Li K, Laliberte N, Montaner JS, Schechter MT (2002). Opportunities for prevention: Hepatitis C prevalence and incidence in a cohort of young injection drug users. Hepatology.

[B44] Clatts MC, Goldsamt L, Neaigus A, Welle DL (2003). The social course of drug injection and sexual activity among YMSM and other high-risk youth: an agenda for future research. J Urban Health.

[B45] Rhodes T (2002). The 'risk environment': a framework for understanding and reducing drug-related harm. International J Drug Policy.

[B46] World Health Organization (2006). Harm reduction approaches to injecting drug use.

[B47] Blankenship KM, Koester S (2002). Criminal law, policing policy, and HIV risk in female street sex workers and injection drug users. J Law Med Ethics.

[B48] Shannon K, Kerr T, Allinott S, Chettiar J, Shoveller J, Tyndall MW (2008). Social and structural violence and power relations in mitigating HIV risk of drug-using women in survival sex work. Soc Sci Med.

